# ‘The beauty and the less beautiful’: exploring the meanings of dying at ‘home’ among community and practitioner representatives and advocates across Canada

**DOI:** 10.1177/26323524231156944

**Published:** 2023-03-14

**Authors:** Laura Funk, Marian Krawczyk, Maria Cherba, S. Robin Cohen, Carren Dujela, Camille Nichols, Kelli Stajduhar

**Affiliations:** Department of Sociology and Criminology, University of Manitoba, 307-183 Dafoe Road, Isbister Building, Winnipeg, MB R3T 2N2, Canada; School of Interdisciplinary Studies, University of Glasgow, Dumfries, UK; Department of Communication, University of Ottawa, Ottawa, ON, Canada; Lady Davis Research Institute, Montréal, QC, Canada; Institute on Aging & Lifelong Health, University of Victoria, Victoria, BC, Canada; Department of Sociology and Criminology, University of Manitoba, Winnipeg, MB, Canada; School of Nursing, University of Victoria, Victoria, BC, Canada

**Keywords:** dying at home, hospice care, interpretive research, palliative care, place of death, public preferences

## Abstract

**Background:**

Significant structural and normative pressures privilege the ideal of dying at home in Canada. At the same time, the social complexities and meanings associated with dying in particular locations remain critically unexamined.

**Objective:**

The aim of this study is to explore how diverse community members, including health and social care stakeholders, talk about preferences for locations of dying, with a particular focus on meanings of dying at home.

**Design:**

Semi-structured virtual interviews were conducted with 24 community and practitioner representatives and advocates across Canada during the Covid-19 pandemic. This included compassionate community advocates, palliative care professionals and volunteers, bereaved carers, and members of queer, rural, and immigrant communities. Participants were asked about their own preferences for location of dying and elaborated on these aspects with regard to their client population or community group.

**Results:**

Our analysis illuminates how meanings of dying at home are connected to previous experiences and perceptions of institutional care. As such, participants’ perspectives are often framed as a rejection of institutional care. Dying at home also often signals potential for preserving ontological security and relational connection in the face of life-threatening illness. However, participants’ expertise simultaneously informs a sense that dying at home is often unattainable. At times, this awareness underpins interpretations of both preferences and choices as contingent on considerations of the nature and type of illness, concerns about impacts on families, and available resources.

**Conclusion:**

The ideal of dying at home is nuanced by identity, relational, and structural contexts. Knowledge from this study can inform realistic and practical person-centered planning across care settings. It can also help create more representative public policy and health system quality indicators regarding a ‘good death’ that do not rely on or perpetuate undeveloped and unrealistic assumptions about dying, home, and family care.

## Introduction

In 2015, Statistics Canada recorded that almost two-thirds (61%) of people in Canada died in hospital settings, compared with 15% of people who died in their own home the same year.^1^ At the same time, Canada, similar to many other predominantly English-speaking countries, has seen a gradual increase in home death rates since the 1990s.^1–3^ This increase coincides with structural pressures and individual preferences toward aging and dying in place, including, but not limited to, early hospital discharges, health care consumerism, and the legalization of medical assistance in dying. While vital statistics data between 2007 and 2019^3^ suggests a lower overall proportions of home deaths than Statistics Canada, both sources confirm the trends toward decreases in hospital deaths and gradual increases in home deaths. Contributing factors in variations between provinces, communities, and individuals regarding location of death include relatively low or varying access to palliative care supports and home care,^4^ personal wealth and social capital, and community and family resources.^3^

Research with family caregivers has found some idealization of dying at home within their interactions with professionals.^5,6^ Tied to conceptions of a good death, this idealization is also prominent in palliative care philosophy and professional guidelines, and among practitioners.^7,8^ Policy documents also tend to posit home as the best place for death and dying.^9–11^ In Canada, dying at home is institutionalized as a health system quality indicator,^12^ reflecting how ‘palliative care services often consider the achievement of home care and home death as an outcome measure’.^13^ Moreover, there is growing attention to the role of community networks in care for persons who are dying at home, in part due to the growing popularity of ‘compassionate community’-based approaches.^14^

Research delineates the importance of feelings of comfort, safety, belonging, familiarity, autonomy, privacy, and quiet at the end of life, which are all normative characteristics of privileged home settings.^15–19^ These and other insights about the desire for dying at home come from research primarily conducted with family caregivers, older adults, or persons diagnosed with terminal conditions.^15,20,21^ However, not everyone may want a home death. People who may be less likely to want to die at home include low-income persons,^22–25^ widows, those living alone or single,^22,25^ and older persons.^22,26–29^

Some research provides insight into why people may not want to die at home, highlighting conditional considerations such as practical realities, safety and quality of life, medical management or pain and symptom control, protecting family, uncertainties related to terminal illness, fear of potentially dying alone, and wanting to put trust in professionals.^28,30–35^ Although existing research examines how structural inequities shape meanings of dying and care in some disadvantaged groups – invoking feelings such as isolation or anxiety,^15,17,20,21^ how structural forces shape perceptions of and preferences for, as well as interpretations of the meaning of dying at home for those in these groups, needs to be more fully explored. Moreover, the associative meanings of institutional and hospital care at the end of life are less often explicitly explored (with some exceptions).^30^

Overall, while research has documented and assessed preferences for and experiences with dying at home, the associative and symbolic meanings of dying in different locations, as well as the interpretive processes involved in meaning-construction in this regard (including the role of socio-cultural narratives or ‘scripts’), need further study.^36,37^ Various social meanings, social relations, and actions about family care, service use, and the meaning of ‘home’ shape our thoughts and feelings about death and dying in complex ways.

Consequently, there is need for further exploration not just of preferences and perceptions of dying at home, but of what dying at home means or represents for different people, and why. The purpose of this article is to explore the nuanced complexity of meanings of dying at home among diverse community members, including health and social care stakeholders, as these meanings manifest in their talk about preferences and logistics of dying at home.

## Methods

### Participants and recruitment

This project was part of a larger multi-method Canadian study exploring public preferences for and meanings of dying in different locations.^38^ Within that project, we interviewed a group of stakeholders whose perspectives are less often integrated into research on dying at home, which focuses more on the perspectives of (often homogeneous samples of) dying patients and their physicians. We sought both professional and non-professional community members who, due to their frequent interaction with marginalized or diverse subgroups or experience acting as a group advocate or representative, worker, or volunteer with these groups, were well suited to speak to these groups’ specific concerns. Groups were theoretically sampled based on what research indicates might be sources of variation in preferences and abilities to die at home: rural older adults, immigrant populations, French-Canadians, former family carers, Two-spirit, lesbian, gay, bisexual, trans and queer (2SLGBTQ+) adults, and marginally housed persons.^22,25,27^ Participants also included care providers with experience caring for dying persons and volunteer members of the ‘compassionate communities’ network.^14^ Twenty-four participants were recruited both through the research team’s professional and social networks and through snowball sampling, primarily in but not restricted to the urban areas of Montréal, Winnipeg, and Victoria. This process, and the interviews, occurred during roughly the first year of the Covid-19 pandemic in Canada (summer 2020 to summer 2021).

What emerged, sometimes actively during interviews, was that several participants often spanned multiple of the above-mentioned roles, experiences, and identities. As such, although participants were primarily recruited based on professional or advocacy roles in terms of populations they work with [compassionate communities (*n* = 4); professional palliative care service provision (*n* = 6), volunteer palliative care service provision (*n* = 2), service provider/advocate for inner-city, structurally vulnerable populations (*n* = 4); service provider/advocate for 2SLGBTQ+ communities (*n* = 3); service provider/advocate for rural communities (*n* = 3); representative/advocate for immigrant communities (*n* = 2)], participants’ talk about their own preferences was also informed by their personal experiences as bereaved family caregivers (*n* = 7), being 2SLGBTQ+ (*n* = 2), having immigrated to Canada (*n* = 2), residing in a rural area (*n* = 4), or identifying as French-Canadian (*n* = 6).

### Data collection

Semi-structured qualitative interviews were conducted and recorded over Zoom, using a key informant style approach.^39^ Interviews averaged 67 min in length (range, 36–130 min). Four trained research assistants and two team investigators conducted interviews in either English (*n* = 22) or French (*n* = 2) and helped with transcription and analysis. Questions (Supplemental Appendix A) elicited interviewees’ beliefs and meanings surrounding dying and care responsibilities in different locations, and, if they were involved in advocacy and service provision for diverse or marginalized persons, their perceptions of the meaning of dying at home for these persons. French language transcripts were translated into English for analysis.

### Analysis

The methodological approach informing the analysis draws on social phenomenology^40^ insofar as we explored not only the manifest content (e.g. what participants state that dying at home means to them, in response to a direct question) but also the subtler and symbolic meanings revealed in participants’ talk (e.g. as revealed through metaphors, everyday features of speech, emotional responses, expressions of uncertainty, appeals to dominant-normative frameworks, use of contrasts). Transcribed data were analyzed by the first author, first through a process of overall familiarization with the data, followed by a categorizing and organizing of interview content that conveyed particular meanings associated with dying in different locations (and care in this regard). It was not a goal of analysis to examine causal associations or patterning between participants’ group or identity membership and their [categorized] perceptions or preferences. Rather, the goal was to analyze the complexity and nuances of meanings and interpretations of dying at home within and across a diverse sample of lay and professional stakeholders. This process of organizing meanings in the data informed the development of conceptual themes. All team members, including three that conducted and transcribed interviews, provided ongoing analytic insights into the data analysis, which was led by the first author. Researchers’ collective feedback (verbally and in writing) helped the analyst reconceptualize findings and refine themes at multiple points, resulting in a more sophisticated and theoretically informed analysis using an iterative process. Preliminary summaries were also sent to participants and presented to 27 key stakeholders across Canada at an invited workshop; both participants and stakeholders had opportunities to comment on the overall findings and validated main analytic themes and their relevance to various provinces.

In this article, we focus on three themes related to meanings and interpretations of dying at home. First, how identities and social positions are expressed and reinforced in talk about the idea of dying at home. Second, how the caring relations in specific places and spaces are expressed and further reinforced in talk about dying in these places. Third, how interpretations of dying at home were shaped by participants’ understandings of contextual and structural realities beyond individual control [available formal supports and expertise in community, especially as this intersects with end-of-life (EOL) symptoms and conditions, and with implications for family].

## Findings

### Theme 1: ‘Home is a history of who I am’: identity expression in talk about dying

The meaning of, and in some cases preference for, dying at home was tied to identity. People’s expressed preferences for locations of dying and their interpretations of the meaning of home reflected and reinforced their selfhood. For instance, when actual physical spaces, locations, and objects were important for some people’s visions of dying at home, this was connected to both their identities and structural circumstances. With reference to how care provided in institutional environments can erode selfhood and autonomy, participants often conveyed that dying at home preserved selfhood toward the EOL. This also manifested in participants’ intertwined use of concepts of familiarity, comfort, control, and personalization, including being able to have something familiar around them at the EOL, that helps them feel most like themselves. Subthemes illuminate two key aspects of this phenomenon: (a) identity-support as safety and (b) the importance of everyday choices and control.

#### Identity-support as safety

Assumptions about home as safe were connected to some participants’ previous experiences and life histories in other settings. One bereaved family caregiver, for instance, expressed:

Home is a place of safety, also the place where I feel nourished and where I can get quiet. For me home is a refuge and a place of recharging. I was an only child so I’m used to having a lot of my own space to sit.

Participants often connected ‘home’ to familiar objects (collections, plants, photo albums, books, nostalgic items from loved ones, etc.) that provide identity signals and thus comfort:

You have all those things that remind you of who you are, more than just this tumour or . . . this heart disease. You’re a whole person and home helps to remind you of all those aspects of who you are, the walking sticks in the corner or whatever . . . you still have the visual cues that remind you there’s an integral whole person inside, inside this shell that’s wasting away. It’s like history. Home is history of who I am. (Palliative care professional)

If I had the choice . . . I would prefer to die at home just for the simple fact that I find more comfort at home than anything . . . just being around everything that I’ve gotten and accumulated over the years, junk or no junk, than anything. (Rural resident and community resource coordinator)

Being around what is familiar (and feels safe) may be particularly salient in a context in which, as many participants emphasized, most people are themselves largely unfamiliar with dying.

Although participants’ connections of home to familiarity and safety can be interpreted as reflecting their relative privilege, an emphasis on protecting selfhood can be especially important for those whose identities are constantly under threat. Some participants expressed that persons who have had negative and stigmatizing experiences in public spaces and health care settings may prefer to die at home, to protect their identities (and bodies) from harm in public spaces. For instance, one participant commented on the need for 2SLGBTQ+ persons to be safe from traumatic and uncomfortable experiences. This was a reflection not only on institutional settings, but on the broader context:

Some people have just been traumatized too much by being out in public or being assaulted or victimized . . . so . . . For some of them, being at home means being able to be safe or be yourself until the point that you pass on. (2SLGBTQ+ service provider)

#### Everyday choices and control

That being at home can facilitate control over simple everyday choices and circumstances when dying was also closely tied to identity and previous experiences both of home and institutional environments. One former caregiver and volunteer stated,

[Home is] where I get to decide how I live. . . . it’s a place where I care for myself and I also have a relatively comfortable level of control, autonomy and self-determination within my home.

Other participants likewise prioritized choice, in home environments, over what to eat, the numbers and kinds of visitors, and routines. As one volunteer and former caregiver commented,

If I want to have the flowers come in, or to have a certain food – it seems like I’m a bit of a foodie here – but I’m thinking that those things are to me, the meaning, and if I want to have three people come visit or I don’t have anyone visit, it is a control of that . . .

Another participant, however, emphasized efforts made by hospices with regard to control: ‘[you can] wear your real clothes. Allow people freedom to eat when they want, or go to bed when they want . . .’ (palliative care provider).

Life experience and social status shape identity in complex ways. In turn, identities both shape and are expressed and reinforced within talk about preferences for place of care and dying. Participants tied dying at home to issues of individual identity, often but not always as a direct rejoinder to identity threats associated with care in institutional settings. Yet participants’ comments in this regard rely on and reinforce particular assumptions about what is possible with the home environment in contrast to other settings. There was also no talk about how receiving formal services in the home environment might reshape homes in particular (e.g. medicalized) ways or constrain choice and control of everyday routines.

### Theme 2: the caring relations in spaces and places before and after death

Meanings and preferences related to locations of dying were embedded in participants’ experiences with and perceptions of the types of care and the caring relations associated with various places. Participants defined their preferences for dying at home against other spaces/places that were *not* home. One palliative care professional reflected, ‘dying at home . . . it’s in opposition to what? There’s often this paradigm, we want to die at home to avoid dying in the hospital or in the emergency room or in an intensive care unit’. Subthemes below draw out three particular aspects of caring relations that were drawn out through such contrasts: (a) being treated with dignity and respect; (b) being with or near friends and family; and (c) being embedded in a caring community.

#### Being treated with dignity and respect

Through contrasting spaces, participants implicated particular types of care and, in particular, types of caring relations. A compassionate community advocate expressed that dying at home would mean appropriate care: ‘what is not going to be at a busy hospital or an emergency room or being in some sort of institutional place that’s not geared for the process of dying’. Particularly among compassionate community advocates, dying at home symbolized reclamation of a natural approach to and acceptance of dying, in contrast to the ‘therapeutic relentlessness’ of institutional or medical care, as well as ‘inappropriate’ and curative-focused intervention. Discussion of non-curative focused care in hospices and palliative care units was often absent in these narratives, with the main contrast being between hospitals and home.

However, other participants (most notably, those from palliative care backgrounds) had witnessed more positive dying and care in institutional contexts, especially palliative care units. In turn, participants’ observations of good EOL care in institutional settings tended to inform broad conceptualizations of dying ‘at home’. For instance, one former family caregiver noted that although they would personally prefer to die at home, this did not necessarily refer to any particular physical place. Their mother died in a long-term residential care facility after living there for a decade, and this participant characterized her mother as having died a good death ‘at home’ since they were able to make her room a cozy and familiar space, and familiar staff and her family were present when she died. Another compassionate community advocate referred to helping a friend who wanted to die at home, but eventually died with good care in hospice. They indicated how this experience helped them personally develop an expansive definition of home:

There’s common kind of feelings of what is home – safety and comfort and that kind of thing – and so that’s what I would be looking for; whether or not that happens in a home setting or that happens in a hospice, or that happens in another place. To me it’s more about how you are treated and that there’s the dignity and respect.

Fundamentally, a preference to ‘die at home’ signaled a desire for a particular kind of caring relations, often informed by prior experiences in particular spaces. Reference to dying at home as reflecting and further reinforcing social integration and relational connection to family and community was also often juxtaposed by participants against an unwanted alternative of neglect and isolation. Below, we illustrate how this signaled a desire for relational integration with others.

#### Being with or near friends and family

For nearly all participants, dying ‘at home’ meant being with and near loved family and friends (including chosen family) toward the EOL. One rural resident and service provider expressed that rural older adults prefer to remain in their own home, because ‘home, to [the seniors I work with], is family. That’s what it means’. This was generally conveyed as a long-standing concern, not unique to the Covid-19 pandemic and associated institutional lockdowns. However, one participant noted how the pandemic infused decision-making around place of dying with a heightened fear about being separated from loved ones.

One 2SLGBTQ+ young adult, who moved frequently since leaving their family of origin and does not own their home, preferred to die where most of their family lives:

. . . at my family’s house and the place where my parents and my siblings live . . . most of my family lives close together except for me, so being there, so that I had access to my family and all of that.

Another participant, a first-generation immigrant from India, had a difficult bereavement after the death of her mother in India, as she was unable to be present. This informed her personal emphasis on being at home when dying, because she believed this would ensure her husband could be present at the death: ‘I would love to die with that feeling that I was loved where I was last’.

In this regard, participants often critiqued hospital visitor policies (even pre-pandemic) and the logistics, physical layout and size of rooms in institutional settings. One Muslim community member had experienced hospitals as unwelcoming to visitors, not only because of the constrained physical environment, but because of subtle symbolic interactions that convey that visitors do not belong (‘every time they walk by they said, “excuse me” like I was a guest’). These participants’ cultural and religious traditions required more than small group visits or family involvement in care; rather large numbers of community members should attend a dying person, for long periods of time. Based on their experience, this was not possible in hospital (though could be possible in a palliative care unit).

#### Being embedded in a caring community

Compassionate community advocates further positioned dying at home as signifying and contributing to broad reintegration of aging and dying persons in communities. For at least one of these participants, their personal situation (having no nuclear family of their own) motivated them to ‘be creative’ in nurturing community for themselves and, in turn, for others. Other participants expressed finding purpose through building community aid for dying persons, regarding this as opportunities for others to discover similar meaning. For instance, one participant described a neighborhood that rallied around a low-income woman without family who wanted to die at home, noting how neighbors gained a sense of purpose, community, compassion, and commitment. One member of the 2SLGBTQ+ community believed that dying at home could be supported in this community, invoking this historical example:

If you take a look back [. . .] at the HIV/AIDS crisis, and how it was the lesbians who took care of the brothers, and they were the ones who, kind of dropped everything that was going on in the 80’s and . . . provided that care.

The Muslim participant cited earlier wished their own neighborhood could become more connected and better respond to and support dying and bereaved persons (they acknowledged Covid-19-related barriers in this regard). They also noted how a desire for being closer to extended community (and a converse fear of isolation) was a reason that dying members of their cultural-immigrant population sometimes return to their country of origin (in their words, back ‘home’). Integration, in this sense, was about not only ‘being supported’ *per se* but about relationality – being around people who care about you. Since culturally the importance of integration extends after death, it was also important to have people who can visit your grave to maintain your memory. Nuancing this general theme of integration in a caring community, a few participants expressed that ‘feeling part of the world’ and surrounded by others could also occur in institutional settings. These participants referenced how this may prompt a desire to die in such settings, especially for those isolated at home, such as rural seniors without family.

Overall, from one angle, participants’ narratives highlight the problems with EOL care in institutions, and how people experience care in those settings (with palliative care professionals diverging somewhat in this regard). Formal care relations and services in the home environment were, in contrast, presented as relatively unproblematic. From another angle, a more complex picture emerges wherein participants’ preferences for dying at home express their relational identities and desires not only for a particular kind of care (e.g. non-curative) but even more so for particular caring relations. Strong desires to be embedded into caring relations and community both before and after a death support a broad conceptualization of ‘care’ (e.g. being cared about).

### Theme 3: qualified, tentative preferences as responses to contextual realities

When speaking about either their own preferences for location of dying or those of community they served or advocated for, participants frequently emphasized variation, flexibility, nuance, and contingency upon (often uncertain) circumstances. This occurred even for participants that leaned elsewhere in their interviews toward idealized visions of dying at home, but was especially prominent among palliative care professionals, who drew on the complexity of their observations of dying in various settings.

In three subthemes below, we explore how participants’ emphasis on contingency and nuance reflected their awareness of (a) the nature of illness and symptoms, (b) limitations and inequalities in accessing formal services, and (c) potential impacts on family.

#### ‘It depends what I’m dying of . . .’

Participants commonly highlighted flexible expectations and tentative preferences given the uncertainty and unpredictability around what type of life-threatening illness they might develop, and associated symptoms and needs. One former caregiver noted that future circumstances may not align with her tentative hopes to die at home, citing how her grandfather wanted to die at home, but developed dementia, and died in long-term residential care. In this respect, some acknowledged dying at home as more feasible for those with low needs and straightforward symptoms. For complex needs or symptoms, some participants viewed institutional settings as providing emotional security through trusted expertise and professional support. Conversely, the home could be a space of anxiety, if symptoms are frightening:

houses aren’t set up for that and [hospital staff] know what they’re doing with that machinery. And I don’t want to screw something up and be responsible. (Rural bereaved family caregiver)

If severe medical attention is required . . . I would not want to see somebody I love suffer pain because we chose to bring them home, or we can’t help them with pain administration [. . .] sometimes they . . . need medical attention 24/7 and if you can’t provide that level [of care], definitely [dying in hospice may be preferable]. (Immigrant community representative)

In such comments, participants articulated an awareness of security needs at the EOL, which they associated with specialized expertise and support. One compassionate community advocate further articulated how structural contexts interact with type of illness or disability. For instance, they expressed that the needs of persons living with disabilities, especially those with challenging behaviors or communication difficulties, are not well met within community group home models and philosophies. The lack of alternative models of support and housing mean such individuals often end up living and dying in long-term care facilities.

#### ‘Others don’t have the luxury of choosing’

Participants’ perceptions of limited formal home-based palliative care supports, and structural (dis)advantages faced by others in this regard, infused their construction of preferences and choice over location of dying as contingent, nuanced, and needing flexibility, and even as a ‘luxury’. One palliative care professional, for instance, stated that her wish to die at home was not ‘strong’ or ‘ardent’ because it was tempered by their firsthand knowledge:

[. . .] given my work I know that it’s not always possible [to die at home], especially with [. . .] very little [formal services] at home. [. . .] we see the beauty and the less beautiful [in dying at home], but we really see the burden that it can have on the loved ones, even with private care or with a care team in place, we know well that the resources aren’t at the level to be able to allow a dignified end of life for the patient or for their loved ones.

A few participants conveyed their own relative control and privilege in even being able to choose their location of dying. For instance, an inner-city service provider sensed their social position, and ability to choose, gave them the privilege of not worrying about the future:

I know I am someone that is always going to have the resources around me that I have a lot of choice and freedom in making those choices so maybe that’s why I haven’t thought about it [yet] . . .

Participants variously expressed how other people may not have choice when there is insufficient access to formal at-home palliative care or hospices, such as in rural areas or when the dying person lacks financial resources (to address gaps in public services) and a suitable physical/home environment. Several emphasized how dying at home was more feasible for those (including themselves) who could hire (additional) private help, especially to alleviate family burdens.

For participants who worked with structurally vulnerable populations, deep-rooted barriers to dying at home that cumulate over a lifetime and extend beyond the realm of clinical practice and policy were especially evident. One such advocate emphasized that structurally vulnerable persons may have built mistrust in systems based on their life experience and may not have their life set up in a way that facilitates the receipt of traditional home-based supports. Other participants described supportive housing for persons with addictions as highly controlled, with few tenancy rights and policies that would not accommodate people dying with palliative care or informal supports. One service provider emphasized that ‘it’s really hard for people to access home care supports if they’re deemed [an] unsafe environment’ due to substance use or mental health issues in some supportive housing units. From this perspective, dying well at home is a luxury of the wealthy; when dying at home does occur for structurally vulnerable populations, this is often embedded within more tragic and unsupported circumstances and meanings. In-depth knowledge of these structural barriers and contingencies had the effect of nuancing these participants’ talk about the meaning of dying at home, often juxtaposing uncomfortably against some of the dominant narratives discussed above.

Finally, several participants also expressed that the choice to die at home was dependent on the availability of family caregivers; those without family networks or who had past family conflict and trauma would be challenged in dying at home. In the next section, we explain how even for those with family to provide care, considerations for impacts on family still nuance and qualify meanings of and preferences for dying at home.

#### ‘If you are thinking about other people . . .’

Participants’ concerns about how dying at home affects families extended beyond concerns about burdens of care provision to decision-making and ‘witnessing’ or retaining ‘difficult memories’. Participants cited potential impacts on, and responsibility required of, family including role conflict, ‘terrible stress’ and worry, and complicated mourning. For instance, a Muslim community advocate commented that although she personally would hate to be in a hospital, she would never insist on dying at home if what was happening to her at the EOL would stress her family too much. In various other comments about family, dying at home was associated by most participants with isolation/burnout, fear/insecurity, risk and burden (exacerbated during the pandemic), and changed feelings toward the home space. Participants wanted to avoid burdening family with care especially when they lack capacity and coping skills, cannot access public services, or if their condition might require more intense care.

There was also concern for guilt or shame felt by families who cannot support someone’s wishes to die at home. For one palliative care professional, not expressing a strong preference was a relational concern with protecting family members from this possibility. She had observed a patient who insisted on dying at home, but was in ‘agony’ and ultimately had to be hospitalized; their husband believed he had failed. The participant emphasized, ‘and I would never want my family to feel that they had failed me’. This participant had also witnessed good care provided in a palliative care unit, and after speaking to this in the interview, added: ‘so . . . as long as we’re being cared for and tended to properly, I don’t think we need to put that pressure on our family to feel like they may have failed if they’re not able to fulfill our wishes’.

Several participants spoke of how they would try to mitigate potential impacts on their families, through saving up money to top-up publicly funded home care services, or moving to institutional settings. One even mentioned this as a reason they might engage a Medical Assistance in Dying (MAiD) program. Another participant described her former (very overwhelming) experience caring for her husband who died at home. As such, not burdening her children with her own EOL care was particularly important for her, and she was open to the possibility of hospice (as were others, for similar reasons). For these participants, expressing preferences for dying in an institutional setting was also a way to express caring and relational identities.

As a few participants acknowledged directly, preoccupation with not ‘burdening’ others may be a manifestation of individualistic North American or Western values. However, at times, participants countered cultural ideals about EOL preferences as individual decisions, by emphasizing relational conversations and decision-making:

I would want to allow [my family] the opportunity to say no to [me dying at home]. . . . we prioritize a lot in the healthcare system, the patient above all else, but to me it’s not just the patient, it’s everyone who experiences this situation . . . so if it’s not something that they are comfortable doing, I completely respect that. (Palliative care professional)

I’ve stated [dying at home] as my preference but I’d also want to make it clear to my loved ones that I can be content wherever. It will be up to each and, because of the condition I’m in and I realize – because we don’t know. And I do not want to overburden my family, so . . . (Compassionate community advocate/bereaved family caregiver)

One advocate for the 2SLGBTQ+ community explained how their perspective changed after conversing with their partner. They previously would have preferred to not remain at home because of the stress and pressure this would entail for their partner and family:

when [partner] and I started to talk about this . . . [partner] got upset with me, how could I be so selfish as to deny her those last few days or weeks together? And, ‘you’ve got to be kidding me, you’re worried because you’re going to be sick and dying, and you’re feeling what? Guilty? About how that’s going to affect me?’ So, now I can see . . . we would find a way to get a healthcare aide in here, we would find a way to get a doctor to visit if necessary, we would find a way to get space in our home, for the bed, and the equipment, and . . . whatever else. And only resort to leaving our home as a last resort.

This participant’s perspective is bolstered by financial security and resources and by their strong concerns, raised elsewhere, about being stigmatized within institutional settings.

In sum, participants’ understandings of contextual realities were anchored to the lack of availability of formal supports and expertise at home, especially as this intersected with potential EOL symptoms and conditions, or had implications for family. Although individualistic concerns around not burdening others manifested in participant talk, at times dominant assumptions about individualized EOL decisions were challenged, when participants emphasized relational decision-making with family.

## Discussion

A strength of our study is its in-depth analysis of interpretations and meanings of dying at home in a small group of people with extensive professional and volunteer experiences, as well as those speaking to the concerns of population groups whose abilities to die at home may be constrained. Study limitations include the absence of the direct voices of the most structurally vulnerable populations, as well as somewhat limited cultural diversity, which should be addressed in future research. Since many participants were embedded within palliative care systems or the compassionate communities movement, findings may to some extent more heavily reflect discourses and ideological or political interests associated with these systems.

Our goal is to explore and illuminate complexities and richness of meaning that emerged even in this ‘snapshot’ of talk from a small, non-representative participant group. At the same time, surveys conducted using vignette methods suggest that some complexity also manifests in the more general public, at least in relation to preferences for locations of dying.^38^

Perhaps the most common feature that nuanced participants’ talk about the meaning of dying at home (and their preferences) was a concern about impacts on family. Avoidance of burdening family with care responsibilities might be viewed as a distinctively individualistic concern connected to a fear of dependence in aging Western societies.^41–43^ However, participants also spoke of other potential impacts on family that they were concerned about, beyond care burden, and some participants from non-Western cultural backgrounds also spoke of concerns about care burdens on family.

Findings illuminate the complexities that emerge when people are asked to elaborate on the meaning of dying in different physical locations, including home. Meanings are shaped in part by life experiences and social position, and individual identities are expressed and further reinforced through this talk. Moreover, this talk signaled participants’ relational identities and desires not only for a particular kind of (holistic, non-medical) care but for particular caring relations, before and after a death, supporting a broad conceptualization of ‘care’. Such findings are further supported by a 2018 Welsh survey,^44^ which found that being at home at the end of life was less important to respondents than being surrounded by loved ones.

Participants in the present study often drew on and reproduced assumptions about dominant-normative characteristics of home, as well as what kind of care and caring relations are possible at home in contrast to other settings. Repeated juxtapositions of (desirable) home-based care and (undesirable) institutional care at times blurred potentially important distinctions between hospital acute care units and specialist palliative care units (with exceptions, especially among palliative care professionals), which indicates the need to develop public awareness about palliative care. Participants’ narratives certainly highlight long-standing problems with institutional (hospital) care provision, however, as well as how life course stigmatization and marginalization shape experiences with and responses to particular care locations and relations.

Formal palliative home care was itself conveyed as relatively unproblematic, with exceptions especially among participants who worked with structurally vulnerable populations. However, participants (especially palliative care professionals) did acknowledge limitations of access and availability of palliative home care, and this nuanced their talk about dying at home (and their preferences), as did their awareness of uncertainty of EOL symptoms and conditions.

Knowledge from this study can help unpack common assumptions about the role of place and space of dying, home, and family care within public policy and health system quality indicators.^7,45,46^ It also challenges some systemic assumptions around planning for end-of-life care. For instance, consistent assumptions that patients almost universally prefer to be at home have been used to advocate for an expansion of home-based palliative care resources^4^ and a focus on avoiding institutional care that underpins health care system quality indicators in Canada.^1^ Interpretations of dying at home in such approaches, as well as in policy documents, are grounded in neoliberal conceptions of choice^9^ and do not effectively convey the complexities of place of death preferences in the public^38^ or as expressed by participants in the present study. Whereas the dominant approach in policy and practice is to conceive of place of death preferences as static and individual, a contingent and relational understanding emerges in participants’ talk about a relational meaning of home as being close to others; the importance of social integration of dying persons; and a need to consider, protect, and care for family members.

This Canadian study contributes to scholarship that has explored the meaning of dying at home in other countries. Australian researchers, Collier *et al.*,^47^ for instance, concluded: ‘home is a dynamic concept for people nearing the end of life and is concerned with expression of social and cultural identity including symbolic and affective connections, as opposed to being merely a physical dwelling place or street address’ (p. 695). One UK study^30^ found that palliative care inpatients viewed these units as the ‘best compromise’ option in the face of concerns about protecting their families, and the uncertainty of illness and symptom progression. Another study^17^ also found that home was framed as a refuge from ‘unacceptable alternatives or places considered controlling, or unsafe’ among rural Australians (p. 1579). Participants in the present study who spoke about marginalized and immigrant populations highlighted this as well. Critical reflexivity is imperative when enacting formal care system supports in people’s homes, to ensure it is safe and does not cause more harm.

Public surveys of preferences for locations of death need to be sufficiently nuanced to convey complexity.^38^ Our findings also contribute to emerging critique of advance care planning efforts to infer, prompt, and document choice of patients’ place of death preferences.^48–50^ It is important that in advance planning conversations, professionals do not assume that everyone wants to die at home^44,45^ or even has a home to die in. Finally, our findings suggest a need to build public awareness about and access to hospices and in-patient palliative care, with journalists, clinicians, and not-for-profit organizations continuing to communicate these options.^44^

It has been argued that during the COVID-19 pandemic, dying at home has become more akin to necessity than a choice,^51^ connected to growing aversion to hospitals or long-term residential care environments as sources of contagion and places where visitors have often been restricted. Future research should continue to explore shifts in interpretations of dying at home due to the pandemic, as well as how this shapes public preferences. Our research suggests that even aside from the pandemic, cultural and material forces shape how people experience dying, home, and caring relations in different settings as they navigate tensions between protecting themselves and concerns about not burdening others in the context of varying, often limited access to formal and informal supports in these settings, as well as lifelong material circumstances.

Inadvertently, dying at home narratives in the policy/public sphere can obscure the contingent, relational nature of EOL preferences, as well as inequities shaping experiences of dying at home and whether or not we have a choice. Findings from our Canadian study confirm and extend research in other countries, through an in-depth analysis of the complexity of how community stakeholders and advocates interpret the meaning of dying at home.

## Supplemental Material

sj-docx-1-pcr-10.1177_26323524231156944 – Supplemental material for ‘The beauty and the less beautiful’: exploring the meanings of dying at ‘home’ among community and practitioner representatives and advocates across Canada Click here for additional data file. Supplemental material, sj-docx-1-pcr-10.1177_26323524231156944 for ‘The beauty and the less beautiful’: exploring the meanings of dying at ‘home’ among community and practitioner representatives and advocates across Canada by Laura Funk, Marian Krawczyk, Maria Cherba, S. Robin Cohen, Carren Dujela, Camille Nichols and Kelli Stajduhar in Palliative Care and Social Practice

## Declarations

**Ethics approval and consent to participate:** Institutional REB approvals were received prior to recruitment. Specifically, this research was approved by the University of Manitoba’s Research Ethics Board, Fort Garry (P2019:091), as well as ethics committees at the University of Victoria Human Research Ethics Board (19-0096) and the Medical/Biomedical Research Ethics Committee of CIUSSS West-Central Montreal Research Ethics Board (2020-1963). After the consent forms (provided by email) were reviewed with participants prior to the interview (and they were given a chance to ask questions), participants provided verbal informed consent recorded over Zoom and noted by interviewers in hard copy notes. Out of appreciation and respect for their time and insights, participants were each offered a $50 honorarium, but they were told they could receive this whether or not they completed the interview. The honorarium was also not mentioned in the initial recruitment materials for the project.

**Consent for publication:** Not applicable.

**Availability of data and materials:** The participants have not consented to the release of the qualitative interview transcripts. There are concerns participant transcripts may make them personally identifiable. As such, the qualitative transcripts are not publicly available.
