# Supplementary material for: ‘The beauty and the less beautiful’: exploring the meanings of dying at ‘home’ among community and practitioner representatives and advocates across Canada
Source: Palliat Care Soc Pract. 2023 Mar 14;17:26323524231156944. doi: 10.1177/26323524231156944 (PMC10017957; doi:10.1177/26323524231156944)
Supplement: sj-docx-1-pcr-10.1177_26323524231156944 – Supplemental material for ‘The beauty and the less beautiful’: exploring the meanings of dying at ‘home’ among community and practitioner representatives and advocates across Canada [file sj-docx-1-pcr-10.1177_26323524231156944.docx]

**INTERVIEW PROMPTS (Individual Stakeholder Interviews)**

**Introduction**

You are encouraged to speak freely about your beliefs and feelings about, and preferences for, place of death and responsibility for end of life care. I’ll ask you first about your own perspective, and then ask you to speak as a stakeholder, advocate or representative more broadly.

**Core Questions**

First, I’d like you to think about the place you would prefer to spend the last few weeks of life, if you were facing an expected death from an illness.

- - *Probes:* Can you tell me more about that place? Why is it your preferred place? COVID impact?

Can you imagine a circumstance where the place you had in mind just now would actually NOT be your preferred place to spend your dying days?

- - *Probes:* Can you explain further? Why these circumstances or scenarios? COVID impact?

In this study we have a specific interest in exploring the idea of ‘dying at home.’ Whether or not home is your preferred place to die, can you tell me what it means, to you, to die at home?

- - *Probes:* Who would you want around you? How would you want them to spend that time with you? COVID impact?

When thinking of the place you’d like to spend the last few weeks of life – is it the same place you’d like to actually die? Why/why not?

I’d like to shift our discussion to your thoughts on who is responsible for helping people who are dying at home. Feel free to draw on any personal or professional experiences that may be relevant, if you are comfortable doing so. Thinking first of family, from your perspective, what generally is the responsibility of family to help people who are dying at home?

- - *Probes:* What should or shouldn’t families do for someone dying at home? Why/why not? What does ‘family responsibility’ look like? COVID impact?

Turning next to government - what is the responsibility of government to help people who are dying at home?

- - *Probes:* What should or shouldn’t government do for someone who is dying at home? Why/why not? What does ‘government responsibility’ look like? COVID impact?

What about other groups – does anyone else have responsibility to help people who are dying at home?

- - *Probes:* What should or shouldn’t they do? Why/why not? (If mentioned – what does ‘community’ look like?) COVID impact?

Now I’d like us to return to think about dying in other places (not at home). How does the responsibility of family differ? Of government? Of other groups?

Lastly, I’d like you to think about whether you personally would help a family member or friend who is dying at home [Or, if you have done this – would you do it again?]

- - *Probes:* Why/why not? What do you think that would involve (any expected challenges, rewards etc)? COVID impact?

Before we wrap up, is there anything else you would like to add? Final comments or thoughts?

***For all advocates/representatives/service providers***

I’d like now to ask you to think about the people you work with or advocate for as part of your professional or volunteer role.

- Where is the place you think they would most prefer to die, if facing an expected death from an illness? (*Probes:* Why? Can you tell me more about that place? Is this also where you would hope that they could die? Why/why not? COVID impact?)
- Thinking again of this group, in what circumstances would they NOT want to die in this place? (*Probes:* Can you explain further? Why these circumstances or scenarios?)
- Again thinking of this group:
  - What do you think ‘home’ means for this group, generally (and why)?
  - What does it mean, for this group, to ‘die at home’? (*Probe:* COVID impact?)
  - Is being supported to die at home a possibility for this population? (*Probes:* what does this look like, how might it differ than for other community members not facing the same barriers? Who is around at the EOL? COVID impact?)
